# Inhibition of acid sphingomyelinase reduces reactive astrocyte secretion of mitotoxic extracellular vesicles and improves Alzheimer’s disease pathology in the 5xFAD mouse

**DOI:** 10.1186/s40478-023-01633-7

**Published:** 2023-08-21

**Authors:** Simone M. Crivelli, Zainuddin Quadri, Hemendra J. Vekaria, Zhihui Zhu, Priyanka Tripathi, Ahmed Elsherbini, Liping Zhang, Patrick G. Sullivan, Erhard Bieberich

**Affiliations:** 1 Department of Physiology, University of Kentucky College of Medicine, 780 Rose Street MS519, Lexington, KY 40536 USA; 2 Department of Neuroscience, University of Kentucky, Lexington, KY 40536 USA; 3 Spinal Cord and Brain Injury Research Center (SCoBIRC), University of Kentucky, Lexington, KY USA; 4 Veterans Affairs Medical Center, Lexington, KY 40502 USA

**Keywords:** Imipramine, Extracellular vesicle, Acid sphingomyelinase, Mitochondria, 5xFAD, Microglia, Astrocytes, TNF-α, IL-1α, C1q

## Abstract

In Alzheimer’s disease (AD), reactive astrocytes produce extracellular vesicles (EVs) that affect mitochondria in neurons. Here, we show that Aβ-induced generation of the sphingolipid ceramide by acid sphingomyelinase (A-SMase) triggered proinflammatory cytokine (C1q, TNF-α, IL-1α) release by microglia, which induced the reactive astrocytes phenotype and secretion of EVs enriched with ceramide. These EVs impeded the capacity of neurons to respond to energy demand. Inhibition of A-SMase with Arc39 and Imipramine reduced the secretion of cytokines from microglia, prompting us to test the effect of Imipramine on EV secretion and AD pathology in the 5xFAD mouse model. Brain derived-EVs from 5xFAD mice treated with Imipramine contained reduced levels of the astrocytic marker GFAP, ceramide, and Aβ and did not impair mitochondrial respiration when compared to EVs derived from untreated 5xFAD brain. Consistently, Imipramine-treated 5xFAD mice showed reduced AD pathology. Our study identifies A-SMase inhibitors as potential AD therapy by preventing cyotokine-elicited secretion of mitotoxic EVs from astrocytes.

**Supplementary Information:**

The online version contains supplementary material available at 10.1186/s40478-023-01633-7.

## Introduction

Current treatments for Alzheimer’s disease (AD) mainly provide symptomatic, short-term benefits, without affecting the cause and progression of the disease. Therefore, a major challenge in the AD research field is to develop new therapies that target causative factors of AD for the prevention or delay of disease onset. Several lines of evidence link the sphingolipid ceramide (Cer) to AD pathophysiology. Firstly, global levels of specific Cer species are elevated in AD patients compared to healthy controls [1–4]. Secondly, high levels of long chain Cer (C22 or C24) in plasma are associated with an increased risk to develop mild cognitive impairment or AD [5, 6]. Thirdly, cerebral spinal fluid detection of Cer levels correlates with some forms of amyloid-β (Aβ) in individuals at high risk of developing AD, implying early involvement of Cer in the pathogenesis of the disease [7]. Lastly, in agreement with these observations, enzymes responsible for Cer anabolic or catabolic production are abnormally transcribed or activated in AD brain tissue compared to healthy controls [8, 9]. What remains to be clarified is whether these observations are a causative or a consequent event in the pathophysiology of AD and the druggability of Cer metabolism to prevent AD [10].

One of the key enzymes generating Cer and linked to AD is acid sphingomyelinase (A-SMase), which is a phosphodiesterase that breaks down sphingomyelin to form Cer in the lysosomes and at the plasma membrane of cells. In the brains of AD patients, the expression levels of A-SMase are increased and the enzymatic activity abnormally high [9, 11]. A-SMase is activated by oxidative stress [12] or TNF-α [13], which are produced in response to Aβ peptides by brain-resident cells, such as microglia, in AD [14]. Microglia are the first responders to Aβ peptide oligomers in the brain, by secreting proinflammatory factors including TNF-α, IL-1α and Complement factor C1q, all of which activate astrocytes [15]. In the context of AD, reactive astrocytes fail to provide structural and metabolic support to neurons and produce extracellular vesicles (EVs) enriched in Cer that are taken-up by neurons [16, 17]. Co-transport of elevated Cer with Aβ peptide as cargo in these vesicles seems to have a mitotoxic effect [16, 17]. Therefore, the SM-Cer pathway controlled by A-SMase may be a potential target in AD by reducing secretion of proinflammatory factors [18], thereafter preventing formation of astrocyte-derived mitotoxic EVs. Classic tricyclic anti-depressants (TCAs) such as Imipramine inhibit the activity of A-SMase [19, 20] by displacing the enzyme from its membrane‐bound substrate [21]. This causes the lysosomal enzyme to be degraded at a faster rate [22]. Due to this peculiar mechanism, these pharmacological agents have been defined as functional inhibitors of A-SMase (FIASMA) [23].

In this study, we hypothesized that Cer generated by A-SMase sustains the inflammatory response by controlling the release of TNF-α, IL-1α and C1q from microglia, and subsequently activation of astrocytes to produce of EVs that induce mitochondria-dependent apoptosis in neurons. We found that inhibition of A-SMase in microglia isolated from adult 5xFAD mice reduced Aβ-induced release of TNF-α, IL1-α and C1q, proinflammatory cytokines that induced secretion of mitotoxic EVs from reactive astrocytes. Administration of Imipramine to 5xFAD mice reduced proinflammatory microglia, reactive astrocytes, and neuronal death. Brain derived-EVs of 5xFAD mice treated with Imipramine were less neurotoxic compared to EVs derived from 5xFAD brain treated with vehicle. Our study highlights A-SMase as a target in AD and warrants synthesis of brain permeable specific A-SMase inhibitors as anti-AD therapeutic strategies.

## Material and methods

### Animals

C57BL/6 wild type (WT) and 5xFAD mice were bred in-house. Animals were socially housed under a 12 h light/dark cycle in individually ventilated cages. Food and water were provided ad libitum throughout the study. Imipramine (Sigma-Aldrich, I7379) was dissolved in Dulbecco’s Phosphate-Buffered Saline (DPBS) without calcium and magnesium (Corning, 21-031-CV) and administered intraperitoneally (IP) at the dose 15 µg/g animals given every 24 h. The volume injected per animal was adjusted to 0.2 mL and administered with insulin syringes. The vehicle DPBS was used as a control. Treatment was given for 28 days starting at the age of 2 months to a total of 36 animals equally distributed between vehicle and Imipramine. Experimental groups were divided based on genotype (WT vehicle N = 9, WT Imipramine N = 9, 5xFAD vehicle N = 9, and 5xFAD Impramine N = 9) and balanced for sex. At the end of the experiment, mice were euthanized. Prior to intracardial perfusion with ice-cold PBS, blood was withdrawn. Next, the brain was quickly removed, and one half was dissected into forebrain and hippocampus and used for extracellular vesicle (EV) isolation, western blotting, and lipid analysis. The other half was immersed in 4% paraformaldehyde (PFA) solution (Thermo Scientific, J19943-K2) for cryo-sectioning and fluorescence labelling. All experiments using mice to generate primary cell cultures or drug testing were carried out according to the Animal Use Protocol approved by the Institutional Animal Care and Use Committee at the University of Kentucky.

### Preparation of primary microglia and astrocytes

*Microglia*. Primary microglia were isolated and cultivated from adult WT at 4 weeks of age using MACS® Tissue Dissociation Kit (130-107-677) from Miltenyi Biotec in 12 well plates. Microglia were cultured in DMEM (Corning, 10-013-CV) containing 10% FBS (Atlanta biologicals, S11550) till the day of the experiment when cells were deprived of serum for 24 h. Aβ_1-42_ peptide (AnaSpec, AS-20276) was incubated at 4 °C overnight to allow oligomer formation in serum free media. The next day, 2 µM were added to microglia in presence or absence of 5 µM ARC39 (1-Aminodecylidene bis-Phosphonic Acid, Cayman Chemical, 13,583) or 10 µM of Imipramine. Scrambled Aβ_1-42_ (Anaspec, AS-25382) was used as a control.

*Astrocytes*. Primary astrocytes were isolated and cultivated from adult WT brains at 4 weeks of age using Miltenyi Biotec MACS® Tissue Dissociation Kit (130-107-677) in 12 well plates. Astrocytes were cultured in DMEM (Corning, 10-013-CV) containing 10% FBS (Atlanta biologicals, S11550) till the day of the experiment when cells were deprived of serum for 24 h. 0.5 mL of the microglia conditioned medium was used to incubate astrocytes for 24 h. To characterize EV release in presence of proinflammatory factors, astrocytes were isolated from post-natal mice at day 3 as previously described in T75 flasks [24]. Astrocytes were stimulated with 400 ng/mL of C1q (Abcam, ab282858), 30 ng/mL of TNF-α (Abcam, ab259411) and 3 ng/mL of IL-1α (Millipore Sigma, I3901-5UG).

### Cytokine analysis and A-SMase activity assay

After 18 h treatment with 5 µM of ARC39 or 10 µM of Imipramine, conditioned media from primary microglia were collected and used for detection of secreted cytokines TNFα, IL-1α, (Biolegend, 430904 and 433404) and C1q (Abcam, ab291069) by ELISA following the manufacturers’ instructions.

A-SMase activity was determined in microglia cellular lysate using an A-SMase assay kit (Echelon Biosciences, K-3200) following the company's instructions. After incubation with or without Aβ_1-42_ peptide (rPeptide, A-1163-2) in the presence of 10 µM of Imipramine (Sigma-Aldrich, I7379) or 10 ng/mL of TNF-α receptor inhibitor (MedChemExpress, HY-P9970) or 10 µM of TLR4 Inhibitor (TAK-242), microglia were sonicated in ice-cold lysis buffer containing 150 mM NaCl, 50 mM Tris pH 7.4, 0.6% Triton X-100, and protease inhibitor cocktail Halt™ Protease Inhibitor Cocktail (Thermo Fisher, PI78430). Cellular debris was removed after centrifugation at 10,000 × g for 5 min, and 20 μg protein was used to determine A-SMase activity. A standard curve was calculated following the manufacturer’s protocol and A-SMase activity initially measured in relative fluorescent units (RFU) (see Additional File 1: Fig. S1) was expressed as percentage of control.

### Fluorescent labelling

Microglia, seeded on coverslips, were stimulated with Aβ_1-42_ peptide conjugated to Alexa 555 (AnaSpec, AS-60480-01) overnight. The next day, cells were fixed with ice-cold 4% PFA in PBS for 30 min, permeabilized with 0.25% Triton-X in PBS for 5 min and blocked with 3% BSA in PBS for 1 h. Primary antibodies, anti-Cer IgM (Glycobiotech, MAB_0014) and anti A-SMase (ProteinTech, 14,609-1-AP) were diluted in incubation buffer (0.3% BSA in PBS) at 4 °C overnight. Then, cells were washed 3-times with PBS and incubated with secondary antibodies for 1 h at 37 °C. Coverslips were mounted using Fluoroshield supplemented with DAPI (Sigma-Aldrich, F6057) to visualize the nuclei.

Brain tissue was processed for fluorescence labelling as previously described [25]. Briefly, after perfusion, the half-brain was fixed in 4% PFA solution and then immersed in 30% sucrose/ PBS. Next, brains were embedded in OCT, frozen and cryosectioned at 10 μm thickness (at ~ − 20 °C; Leica). Plaques were visualized with Thioflavin S (Sigma-Aldrich, T1892) while microglia and astrocytes were immunolabeled with goat IgG anti-Iba1 (Novus Biologicals, NB100-1028) or rabbit IgG anti-glial fibrillary acidic protein (GFAP) (Wako Pure Chemical Corporation, Z0334). Astrocytic Cer was detected by co-labelling with mouse anti-GFAP antibody (Santa Cruz, sc-336773, 2E1) and rabbit anti-Cer antibody produced in our laboratory and previously validated. Secondary antibodies were Cy2-conjugated or Alexa-647 donkey anti-mouse IgG, or Cy3-conjugated donkey anti-rabbit IgG, (1:500 Jackson ImmunoResearch). Neurodegeneration was quantified with FluoroJade C  (Sigma-Aldrich, AG325) staining. Images were acquired on a l Nikon A1R Confocal Microscope (Nikon, New York, USA) and analyzed with Nikon NIS-Elements software or with ImageJ for microglia morphology as previously described [26]. Densitometric analysis of the stainings was performed on sagittal brain sections in hippocampus and/or cortical areas at different lateral depth (3–6 sections per animal). Identifiers for control and treatment samples were blinded to the experimenter.

### Isolation of EVs from mouse brains

EVs were isolated from mouse forebrains as we previously described [24, 27]. In short, forebrains were digested in papain for 15 min and a 10 mL serological pipette was used to dissociate the tissue. After initial centrifugation at 300xg for 5 min, the supernatant was subjected to centrifugation at 4000xg (20 min), 10,000xg (40 min) all at 4 °C and the supernatant filtered through a 0.45 µm filter. At this point supernatants from same experimental groups were randomly paired and combined to increase EVs yield (from initial 8 forebrains of WT vehicle treated, 8 5xFAD vehicle treated and 8 5xFAD Imipramine treated we obtained N = 4/group). EVs were collected using the ExoEasy Maxi kit (Qiagen, 76064) following the manufacturer’s instructions.

### Nanoparticle tracking analysis (Zetaview) and Exoview

*ZetaView*. Nanoparticle Tracking Analysis was performed using a Zetaview instrument as previously described [24]. The concentration of EVs was normalized to tissue weight of the starting material, or cell number or cellular protein when EVs were isolated from media.

*Exoview*. Equal amounts of brain- or cell culture media derived EVs were diluted in incubation buffer and loaded on pre-scanned ExoView Tetraspanin chips, placed in a sealed 24-well plate overnight at room temperature. The chips contained spots printed with anti-CD81, or anti-CD9 antibodies or mouse IgG1κ matching isotype antibody, used as a control for non-specific EV binding (NanoView bioscience, EV-TETRA-MI). Chips were then moved to an automated ExoView® CW100 Chip Washer and the tetraspanin program was selected. The following antibody mixture was used to label EVs: anti-Cer IgM (Glycobiotech, MAB 0014) conjugated to Alexa 647 using a kit (Abcam, ab269823), anti-CD81 conjugated to Alexa555 (NanoView bioscience, EV-mCD81-A-555) and anti-GFAP conjugated to Alexa488 (Invitrogen, 53–9892-82), all of them diluted in blocking solution. Chips were then imaged with the ExoView R100 reader using the ExoScan 3.0 acquisition software. Images acquired were analyzed using ExoViewer 3.0 software.

### Cell death and Seahorse assay

Neuro-2a (N2a) were obtained from ATCC (CCL-131™) and maintained at 37 °C and 5% CO2 atmosphere in DMEM supplemented with 10% FBS and 100U/mL penicillin–streptomycin (hyClone, GE Healthcare, UT, USA).

To determine the cytotoxicity of brain-derived EVs, N2a cells were seeded at a density of 10,000 cells/well on 96-well plates and gradually deprived of serum for 1–2 days to allow for differentiation into a neuronal phenotype and reach adequate confluency. Then, cells were treated for 18 h with 10^4^ EVs / cell. LDH release was detected using the CyQUANT™ LDH Cytotoxicity Assay (Thermo Fisher Scientific, C20300) according to the manufacturer’s protocol.

N2a metabolic/bioenergetic profiles were evaluated with XF Cell Mito Stress Assay in a Seahorse XFe96/XF96 analyzer (Seahorse Bioscience/Agilent Technologies, North Billerica, MA). N2a cells were seeded at 10^4^ cells/well in XF microplates (Agilent, 102,416–100) and kept in 5% FBS till the next day when they were deprived of serum and challenged with 10^4^ EVs/cell for 18 h. The media were replaced with XF assay media and a standard mito stress test assay was performed using the Seahorse flux assay. The data were normalized to cell counts using Biotek Cytation 5 (Agilent).

### Western blot

Hippocampus or astrocytes were homogenized in RIPA buffer (250 mM Tris–HCl (pH 7.4), 750 mM NaCl, 5% NP-40, 2.5% sodium deoxycholate, 0.5% SDS) with Halt™ Protease Inhibitor Cocktail (Thermo Fisher, PI78430) by sonication and protein extracts were solubilized with 5X Laemmli sample buffer (10% SDS, 250 mM Tris pH 6.8, 1 mg/mL bromophenol blue, 0.5 M DTT, 50% glycerol, 5% β-mercaptoethanol) and heated to 95 °C for 5 min. Proteins were separated using SDS-PAGE gels (8 or 10%) or pre-cast gels 4–20% (mini-PROTEAN TGX, Biorad) and transferred to nitrocellulose membranes. After blocking with 5% non-fat dry milk (Blotting-grade blocker, Biorad) for 1 h, blots were probed with primary antibodies overnight at 4 °C and incubated with HRP-conjugated secondary antibody. The membranes were developed with SuperSignal™ West Pico PLUS (ThermoFisher, 34577) or SuperSignal™ West Femto Substrate (ThermoFisher, 34095) and images were acquired on a ChemiDoc imaging system (Biorad). For APP and Aβ detection, membranes were probed with 1 µg / mL mAb anti-Aβ (Biolegend, 6E10, 803001). GFAP was detected with anti-GFAP from Wako Pure Chemical Corporation and C3b with ProteinTech (21337–1-AP) antibody. Bands were normalized to β-actin (Santa Cruz, sc-47778).

### Carboxylation and 3-nitrotyrosine measurements

Proteins from the cortex were extracted in Tris-buffered saline containing Halt™ Protease Inhibitor Cocktail (Thermo Fisher, PI78430) by probe sonication. Detection of carbonyls and 3-nitrotyrosine was performed as previously described [28] by immunoblotting using the polyclonal antibodies RbxDNP (Oxy Blot Protein Oxidation Kit, Chemicon-Millipore, Cat No. S-150) and anti-3-NT (Thermo Fisher Scientific, RRID:AB_221457) respectively. Membranes were scanned with a photo scanner (Epson Perfection V600), and slot-blot line densities were quantified by the ImageQuant TL software package (GE Healthcare Bio-Sciences, RRID:SCR_014246).

### Sphingolipid analysis

The sphingolipids of astrocytes, hippocampus and brain derived EVs were quantified by the lipidomics core facility at the Medical University of South Carolina, Charlston, SC (Dr. Besim Ogretmen, director) (https://hollingscancercenter.musc.edu/) as previously described [29, 30]. The following sphingolipids species were determined: sphingosine, sphinganine, sphingosine-1-phosphate, sphinganine-1-phosphate, Cer (N-acyl chain lengths = C14:0, C16:0, C18:1, C18:0, C20:0, C22:0, C24:1, C24:0, C26:1, C26:0). Sphingolipid levels were normalized to protein content or number of EVs measured by NTA.

### Statistical analysis

The statistical analyses were performed using GraphPad Prism version 8.4.3 (686). Unpaired *t*-test was used for comparing of two means or Whitney Mann test when normality distribution was not met. One-way ANOVA followed by LSD or Dunnett's multiple comparisons test was used for comparing more than 2 conditions or for investigating Imipramine effect. Two-way ANOVA was used to analyze lipid measurements in the hippocampus with genotype and treatment as independent variables followed by LSD post-doc test. A *p*-value of < 0.05 was considered significant.

## Results

### A-SMase is activated downstream of TLR4 receptor activation and translocated to the plasma membrane in response to Aβ oligomers

**Fig. 1:**
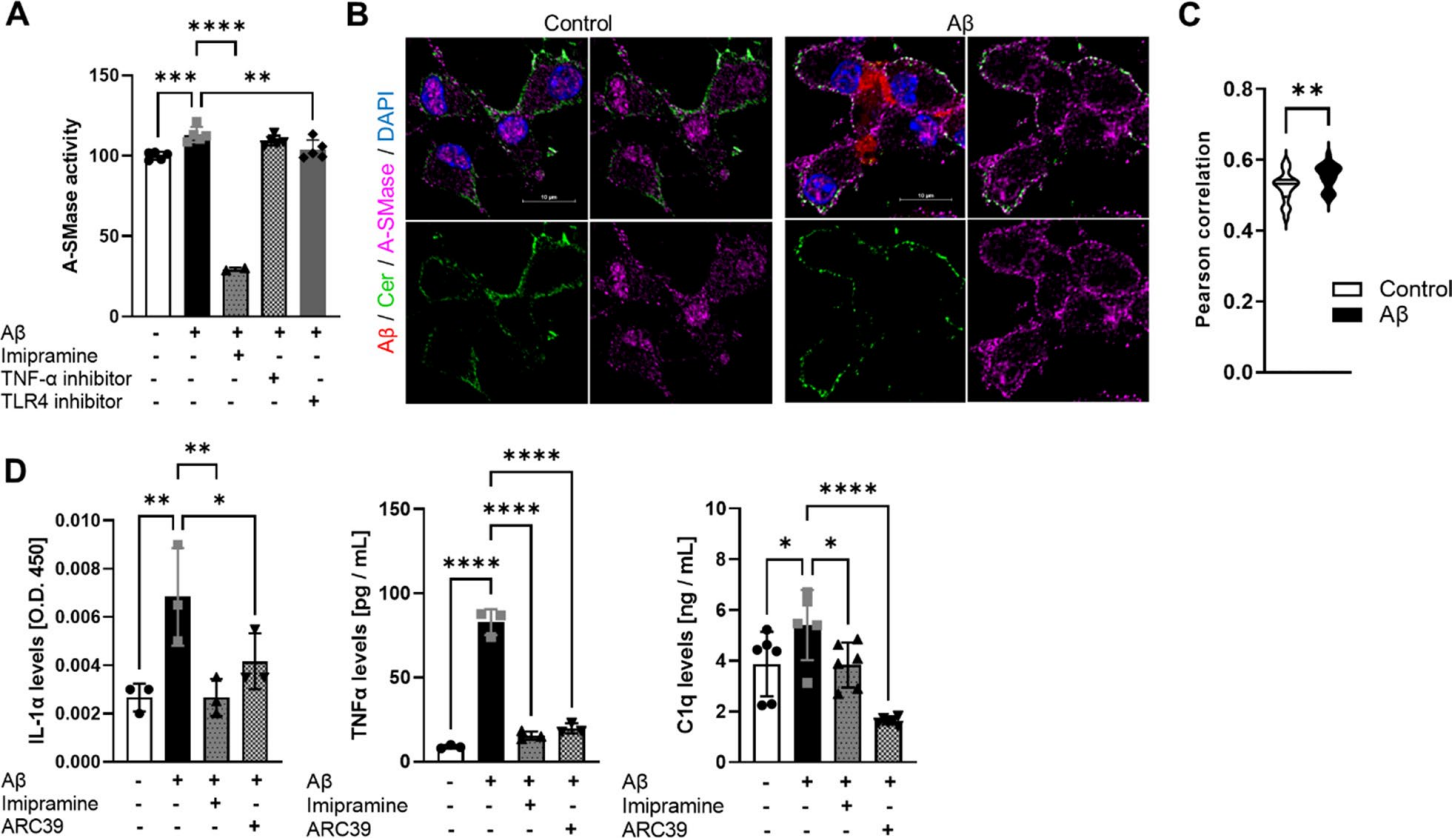
A-SMase is activated downstream of the TLR4 receptor and translocated to the plasma membrane in response to Aβ oligomers. **A** A-SMase activity expressed as percentage of control measured in microglia treated with Aβ_1−42_ oligomers in the presence of Imipramine (10 µM) or TNFα receptor inhibitor (10 ng/mL) or TLR4 receptor inhibitor (10 µM). Bar graphs represent average ± SD of N > 3/group One-way ANOVA, Dunnett’s posthoc testing (**p < 0.01, ***p < 0.001, ****p < 0.0001). **B** Photomicrographs of primary microglia stimulated with or without Aβ_1−42_ peptide conjugated to Alexa 555 (Red) and subsequently immunolabeled with anti-Cer (Green) and anti-A-SMase (Magenta) antibodies. Nuclei were stained with DAPI. Scale bar = 20µm. **C** Violin plots depicting Pearson correlation coefficient between Cer and A-SMase fluorescent intensities of 4–5 photomicrographs / condition. Unpaired *t*-test (**p < 0.01. **D** IL-1α, TNFα and C1q levels measured in supernatant of primary microglia by ELISA after 18 h incubation with Aβ_1-42_ oligomers and of 10 µM Imipramine or 5 µM ARC39. Bar graphs represent average ± SD of N > 3/group. One-way ANOVA, Dunnett’s posthoc testing (*p < 0.05, **p < 0.01, ****p < 0.0001)

A-SMase is found upregulated and more active in brains of AD patients compared to controls [9]. Since it was previously reported that stimulation of immune cells activates A-SMase [31, 32], we investigated if Aβ oligomers could induce A-SMase activity in microglia. Microglia from 4 weeks old wild type (WT) mice were isolated and stimulated with 2 µM Aβ oligomers for 18 h. A-SMase was more active after stimulation with Aβ oligomers (Fig. 1A). A-SMase activation was inhibited by TLR4 receptor or A-SMase inhibitors but not by a TNF-α receptor inhibitor (Fig. 1A), which was tested to exclude TNF-α autocrine activation of A-SMase. Aβ oligomers triggered the translocation of A-SMase to the plasma membrane and increased the co-localization of A-SMase with Cer in the plasma membrane as quantified by Pearson correlation analysis (Fig. 1B and C). Scrambled Aβ_42_ used as a control did not induce co-localization of A-SMase with Cer in the plasma membrane, while aggregates of Aβ_42_ led to a locally increased concentration of A-SMase and Cer (arrows in Additional File 1: Fig. S2A). Importantly, Aβ oligomer-dependent release of IL-1α, TNF-α and C1q was suppressed by ARC39 and Imipramine, a direct A-SMase inhibitor and FIASMA, respectively (Fig. 1D). This data indicates that A-SMase is activated downstream of the TLR4 receptor, and it is translocated to the plasma membrane in response to Aβ oligomers. Furthermore, A-SMase activity is critical for the secretion of IL-1α, TNF-α and C1q.

### Microglia-conditioned medium increases ceramide in reactive astrocytes, which is prevented by inhibition of A-SMase

**Fig. 2:**
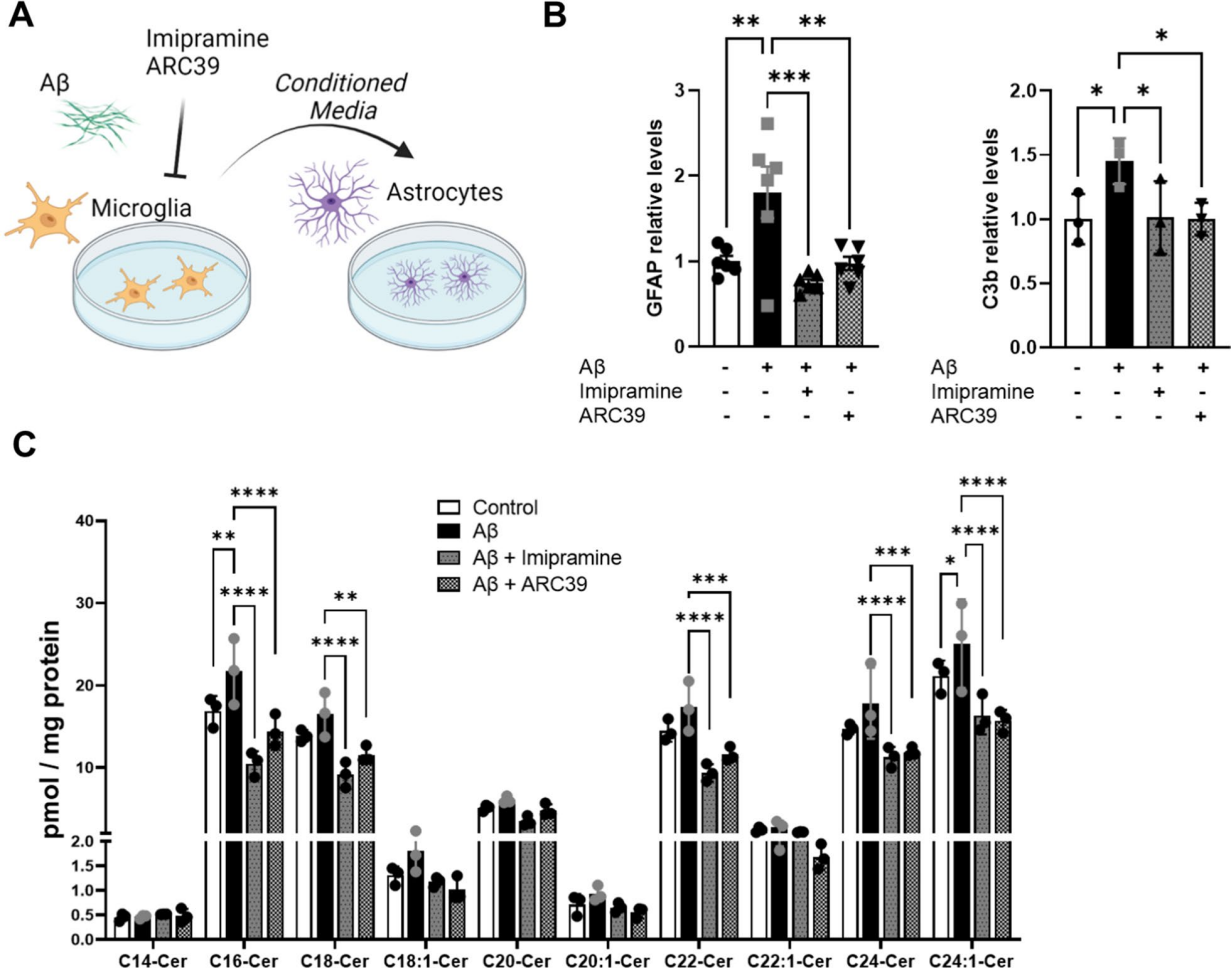
Inhibition of A-SMase prevents astrocytic Cer formation. **A** Schematic diagram showing treatment of astrocytes with microglia-conditioned media. **B** Quantification of GFAP and C3b by western blot analysis in astrocytes after 24 h incubation with conditioned media from microglia (Western blot membranes are shown in Additional File 1.). Bar graphs represent average ± SD of N > 3/group. One-way ANOVA, Dunnett’s posthoc testing (*p < 0.05, **p < 0.01, ***p < 0.001). **C** Quantification of C14:0, C16:0, C18:0, C18:1, C20, C20:1, C22:0, C22:1, C24:0 and C24:1 by mass spectrometry of astrocytes after incubation with conditioned media from microglia. Bar graph represents average ± SD with each N = 3 group. One-way ANOVA, Dunnett’s posthoc testing (*p < 0.05, **p < 0.01, ****p < 0.0001

Since inhibition of A-SMase reduced the release of proinflammatory factors from Aβ-treated microglia we explored whether adding A-SMase inhibitors to microglia would also prevent phenoconversion of wild type astrocytes to an active state, when incubated with medium conditioned by microglia as depicted in Fig. 2A. We did not focus on the contribution of individual factors, but tested the effect of the full range of factors added by microglia to the medium when exposed to Aβ with or without A-SMase inhibitors. As expected, astrocytes incubated with media from Aβ-treated microglia displayed upregulation of GFAP and complement factor C3b, which was prevented when microglia were treated with A-SMase inhibitors (Fig. 2B). Previously we reported that reactive astrocytes in AD show increased levels of Cer in vivo [16, 25, 33, 34]. Therefore, we explored if media from Aβ-treated microglia would trigger astrocytic Cer formation. We found that especially C16 Cer and C24 Cer were upregulated and that treatment of microglia with A-SMase inhibitors suppressed this elevation (Fig. 2C). This data indicates that medium conditioned by Aβ-treated microglia activates astrocytes and increases astrocytic ceramide generation, which is prevented by inhibition of A-SMase.

### The proinflammatory factors IL-1α, TNF-α and C1q*** trigger*** the release of mitotoxic Cer-enriched EVs from astrocytes

**Fig. 3:**
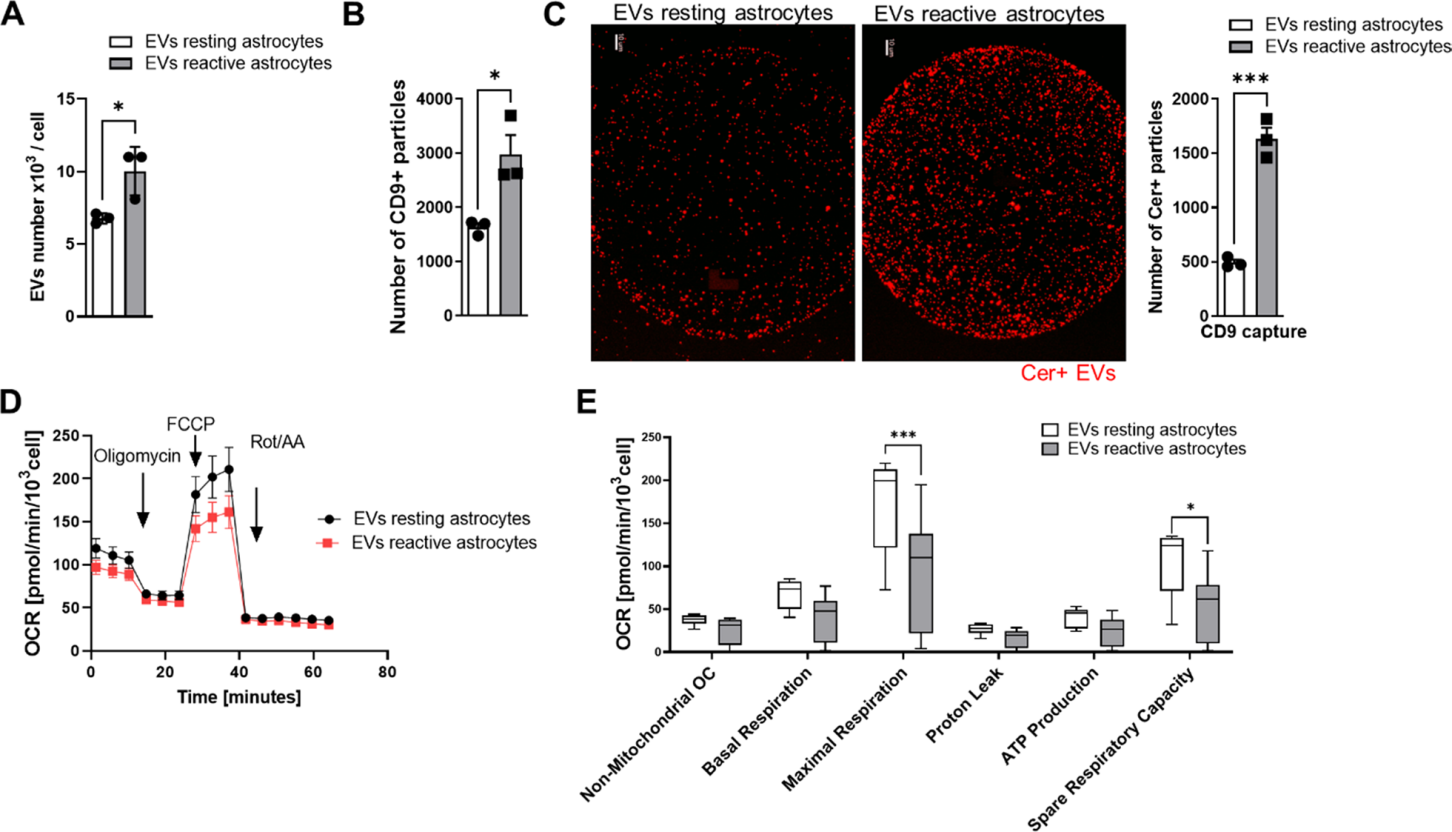
The proinflammatory factors, IL-1α, TNF-α and C1q trigger the release of mitotoxic Cer-enriched EVs from astrocytes. **A** Numbers of EVs per cell measured by NTA after stimulation of astrocytes without (resting astrocytes) or with combination of C1q, TNF-α and IL-1α (reactive astrocytes). Bar graphs represent N = 3/group. Unpaired *t*-test (*p < 0.05). **B** Number of CD9-captured EVs. N = 3/group. Unpaired *t*-test (*p < 0.05). **C** Representative images for Cer positive EVs in CD9 capture spots. Quantification of Cer + EVs. Bar graphs represent N = 3/group. Unpaired *t*-test (***p < 0.001). **D** and **E**) Bioenergetics of neuronal cells incubated with EVs of resting or reactive astrocytes assessed by Seahorse Flux Analyzer using Cell Mito Stress test. Lines represent average ± SD and Box and Whiskers plot of N = 3/group. Unpaired *t*-test (*p < 0.05, ***p < 0.001)

Our data showed that conditioned medium of Aβ-treated microglia contained proinflammatory cytokines, the secretion of which is prevented by the inhibition of A-SMase. It is known that (1) microglia-derived cytokines activate astrocytes [15], (2) cytokines induce secretion of EVs and ceramide generation in astrocytes, and (3) increase of ceramide levels in astrocytes is critical for the secretion of neurotoxic EVs that are enriched with ceramide [35–38]. However, we do not know if proinflammatory cytokines induce secretion of ceramide-enriched neurotoxic EVs by reactive astrocytes. Therefore, astrocytes isolated from P3 pups were stimulated for 24 h with a combination of IL-1α, TNF-α and C1q. EVs secreted by astrocytes were isolated by ultracentrifugation as previously described [24]. Nanoparticle tracker (Zetaview) analysis showed that stimulation with proinflammatory factors increased the numbers of EVs per cell by 1.5 -fold (Fig. 3A). To test if these EVs were enriched with Cer, we used Exoview analysis, a method that immunocaptures EVs by binding to immobilized antibodies against tetraspanins (e.g., CD9) for counting of EVs and single vesicle immunolabeling with different antibodies. The number of CD9 positive EVs was increased by 1.8-fold, consistent with the increase of total EVs as determined by Zetaview analysis (Fig. 3A and B). Immunolabeling or CD9 positive EVs was performed with a fluorophore-conjugated antibody against Cer. We found that the proportion of CD9 positive EVs immunolabeled for Cer increased by 3.3-fold in EVs from stimulated (reactive) astrocytes when compared to EVs from non-stimulated (resting) control cells (Fig. 3B). Since the number of both, total and CD9 positive EVs secreted by reactive astrocytes was increased by 1.3-fold, while the proportion of CD9 positive EVs labeled for Cer was increased by 3.3-fold, the proinflammatory factors must have induced the secretion of EVs that are enriched with Cer.

Based on previous studies showing that Cer-enriched EVs from AD brain target mitochondria [35, 39], we tested if proinflammatory factors induced secretion of mitotoxic EVs from astrocytes. Seahorse analysis showed that the Cer-enriched EVs were mitotoxic to neuronal cells (Fig. 3D and E). This data suggests that IL-1α, TNF-α and C1q trigger the release of mitotoxic Cer-enriched EVs from astrocytes.

### *Imipramine inhibits microglia activation and astrocytic Cer formation *in vivo

**Fig. 4:**
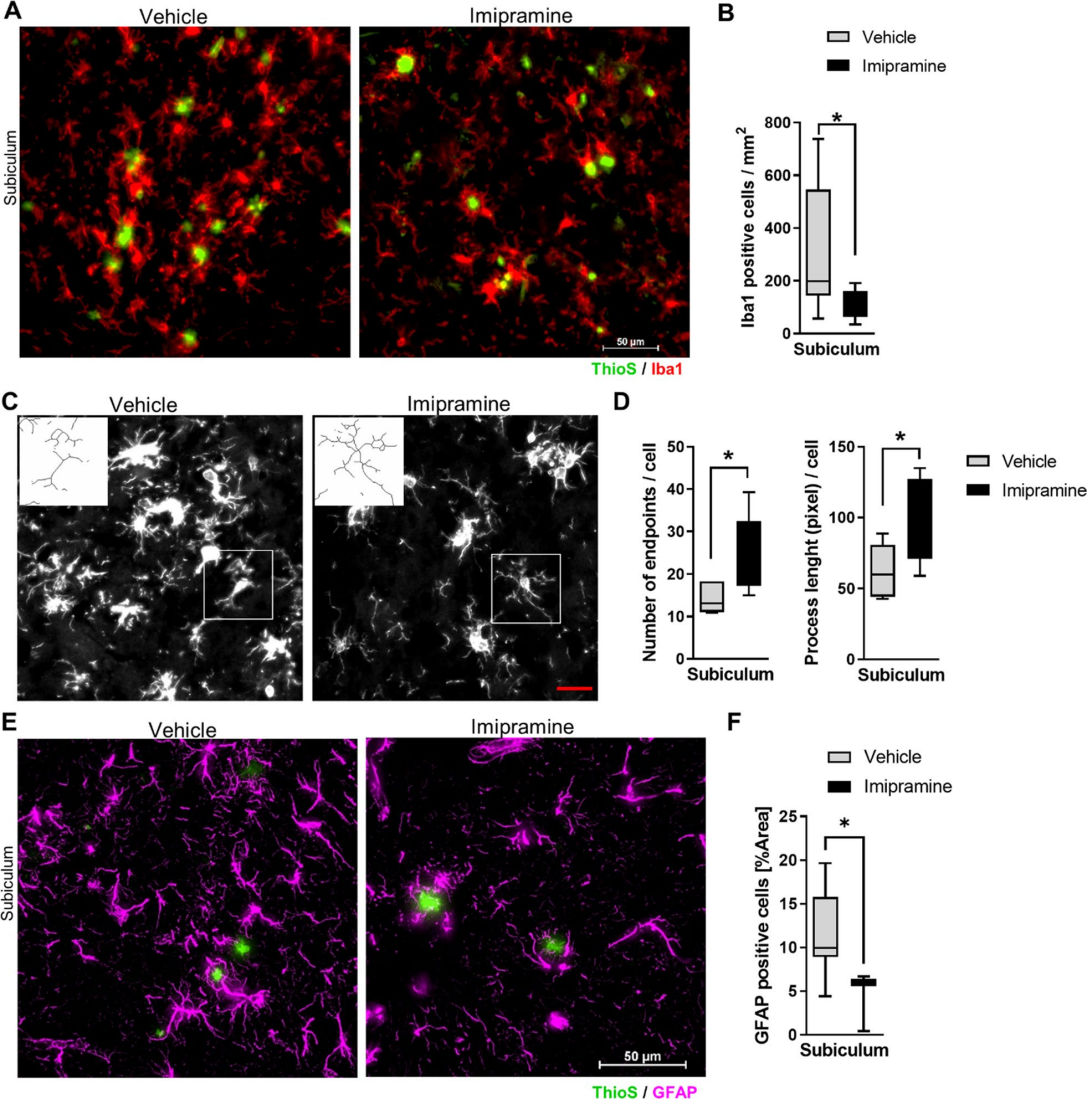
Imipramine inhibits microglia and astrocyte activation in vivo. **A** Representative photomicrograph of Iba1 staining and ThioS in the subiculum region of 5xFAD animals treated with vehicle or Imipramine (Scale bar 50 µm). **B** Densitometric analysis of Iba1 staining represented as number of positive Iba1 cells/mm^2^ (Vehicle N = 5 and Imipramine N = 5 for groups). **C** Illustrations of the microglia morphological analysis applied to a fluorescent photomicrograph captured with 40× objective with a single cell cropped to show details. Scale bar 20 µm. **D** Number of endpoints and length of microglia ramification per cell in vehicle or Imipramine. Morphological analysis was performed on Vehicle N = 5 and Imipramine N = 5 for groups. **E** Representative photomicrographs of GFAP and ThioS in the subiculum region of 5xFAD animals treated with vehicle or Imipramine (Scale bar 50 µm). **F** Box and Whiskers plot of densitometric analysis of GFAP staining represented as a percentage of the area (Vehicle N = 5 and Imipramine N = 5 for groups). Unpaired *t*-test (*p < 0.05)

The anti-inflammatory effects of A-SMase inhibitors in vitro prompted us to investigate whether administration of Imipramine for 4 weeks would affect glial cell activation in an AD mouse model (5xFAD mice). Iba1 is considered a constitutive marker for microglia, which is highly increased in 5xFAD animals compared to WT [40, 41]. Therefore, brain sections were analyzed for Iba1 immunoreactivity. Imipramine decreased the number of Iba1 positive microglia in the subiculum (percentage of area *p* < 0.001) (Fig. 4A-B). We further characterized microglia based on ramifications in the subiculum. The analysis showed that microglia in Imipramine treated 5xFAD mice had more endpoints and longer ramifications (Fig. 4C and D) suggesting an anti-inflammatory phenotype. The anti-inflammatory effect of Imipramine was confirmed by reduced labeling of microglia and astrocytes for iNOS, a marker of activated glia (Additional File 1: Fig. S3A) [42].

**Fig. 5:**
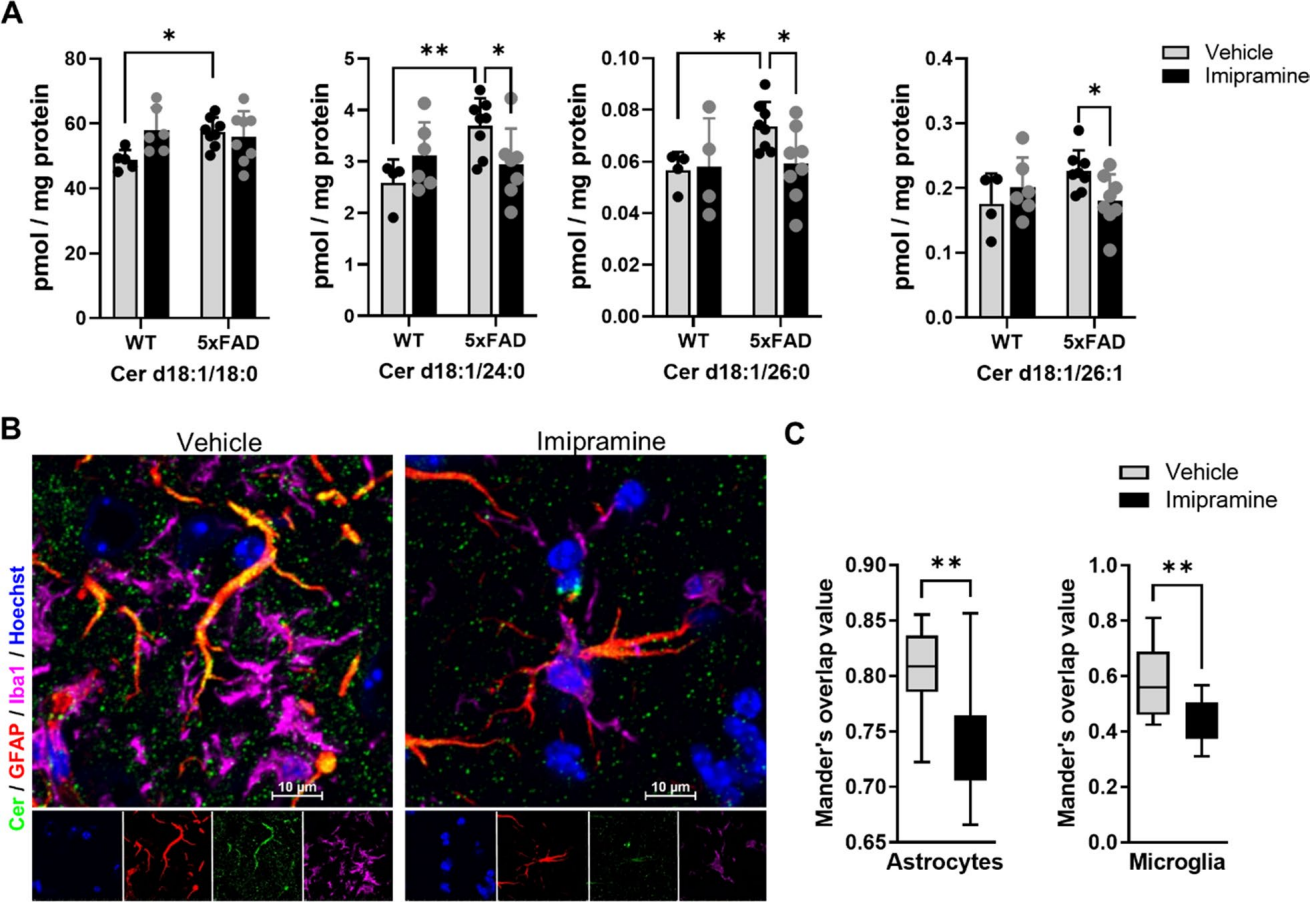
Imipramine prevents astrocytic ceramide formation in vivo. **A **E Quantification of C18:0, C24:0, C26:0 and C26:1 Cer by mass spectrometry in the hippocampus. Bar graph represents average ± SD with each N = 4–8/group. Two-way ANOVA followed by LSD postdoc analysis (*p < 0.05, **p < 0.01). **B** Representative photomicrographs of Iba 1 (Magenta), GFAP (Red), Cer (Green), and Hoechst (Blue) in the subiculum region of 5xFAD animals treated with vehicle or Imipramine (Scale bar 10 µm). **C** Box and Whiskers plot of correlation analysis of GFAP and Iba1 staining with Cer immunolabelling represented as Mander’s overlap values (Vehicle N = 5 and Imipramine N = 5 for groups). Unpaired *t*-test (**p < 0.01)

Next, to determine the extent of astrocytosis in the 5xFAD mouse brain treated with Imipramine compared to control, we performed GFAP immunofluorescence labeling (Fig. 4E). Densitometric analysis showed a reduced area of GFAP-labeled astrocytes in Imipramine-treated mice (Fig. 4F). When investigating the effect of Imipramine on global Cer levels in the hippocampus, we observed that Cer d18:1/18:0 (F test interaction (1, 23) = 5.087, *p* = 0.0339), Cer d18:1/24:0 (F test interaction (1, 21) = 6.499, *p* = 0.0187), Cer d18:1/26:0 and Cer d18:1/26:1 (F test interaction (1, 22) = 4, *p* = 0.0419) were elevated in 5xFAD animals compared to WT and that Imipramine reduced levels of Cer d18:1/24:0, Cer d18:1/26:0 and Cer d18:1/26:1 (Fig. 5A). Next, astrocytic Cer was assessed by immunolabeling as previously described [25]. Co-labeling of GFAP with Cer or Iba1 with Cer was significantly reduced in Imipramine-treated brains (Fig. 5B and C). This data indicates that long chain Cers are elevated early in the hippocampus of 5xFAD and that Imipramine prevents microglia and astrocyte activation and production of Cer by glial cells in vivo.

### Imipramine reduces GFAP positive and mitotoxic brain-derived EVs

**Fig. 6:**
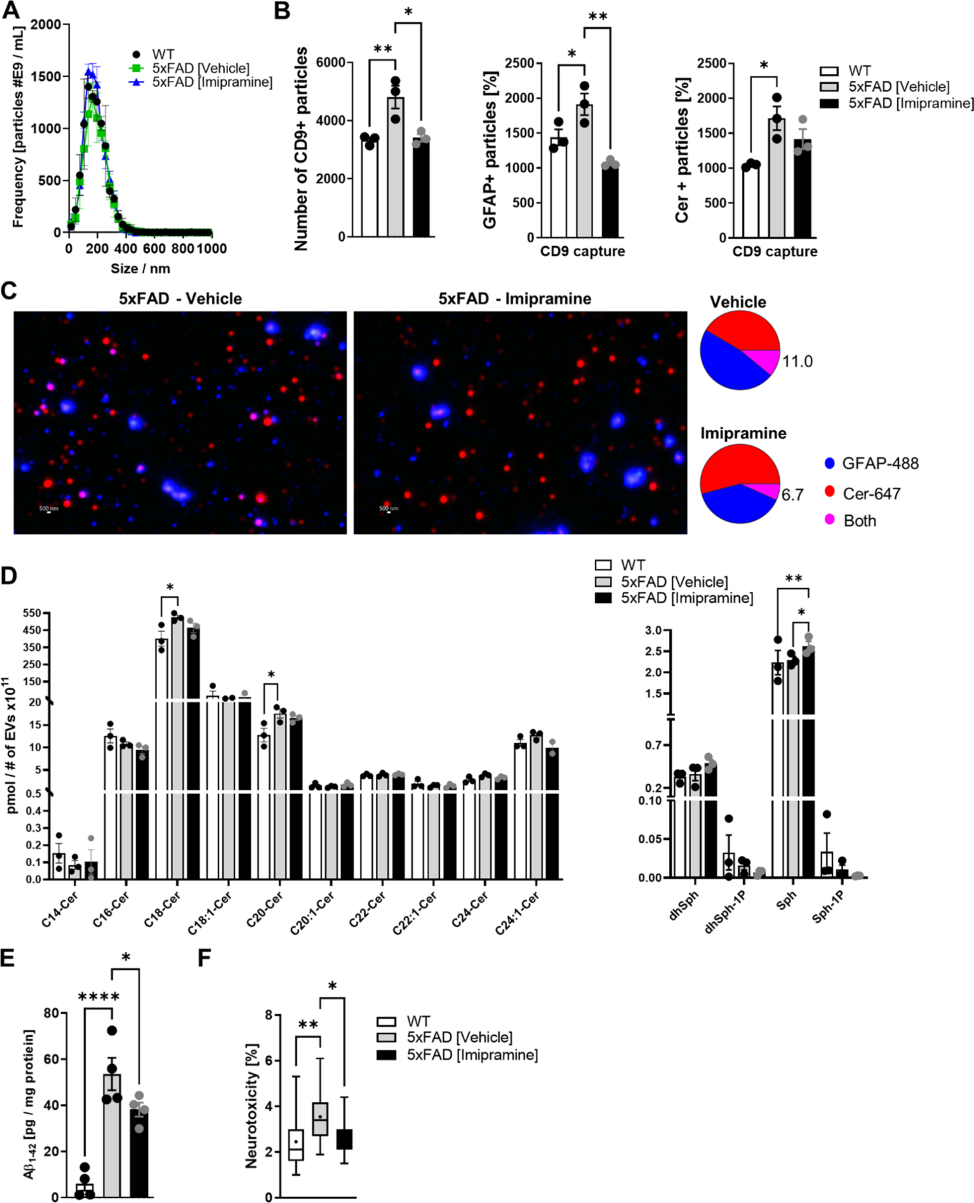
Imipramine reduces ceramide, GFAP, and Aβ levels in 5XFAD brain-derived EVs. **A** NTA measurement of EVs isolated from forebrain normalized to protein content of starting tissue. Bar graph represents average ± SD with each N = 4/group. **B** Representative particle counts on different CD9 capture spots. Counts of GFAP (GFAP+) and Cer (Cer+) positive EVs, CD9 captured. Bar graph represents average ± SD. for three capture spots of N = 4 (pooled)/group. **C** Representative images and pie chart showing percentage of colocalization of GFAP+ with Cer+ in CD9 captured EVs of 5xFAD vehicle and Imipramine treated groups. **D** Quantification of C14:0, C16:0, C18:0, C18:1, C20, C20:1, C22:0, C24:0, C24:1 Cer by mass spectrometry of brain EVs. Bar graph represents average ± SD with each N = 3/group. **E** Aβ_1-42_ quantification in brain derived EVs by ELISA. Bar graph represents average ± SD with each N = 4/group. **F** Neuronal toxicity determined by LDH release after 18 h incubation with brain derived EVs. Box and Whiskers plot with each N = 4/group. One-way ANOVA followed by Tukey postdoc (*p < 0.05, **p < 0.01, ***p < 0.0001, ****p < 0.0001)

Reactive astrocytes fail to provide metabolic support to neurons, and we previously discovered that they release EVs enriched in Cer and Aβ which are mitotoxic [35]. Therefore, we explored if EVs isolated from brain of Imipramine-treated mice showed reduced neurotoxicity. We found that the size and numbers of EVs extracted from the forebrain were similar between WT, 5xFAD vehicle and Imipramine-treated brains (Fig. 6A). However, the proportion of CD9 positive EVs was increased in the 5xFAD animals compared to WT. Additionally, the proportion of CD9 captured EVs, which were co-immunolabeled for GFAP and Cer was elevated in 5xFAD (Fig. 6B). Consistent with our in vitro data with reactive astrocytes (Fig. 2B and C), this data indicated that in 5xFAD mice, the proportion of astrocyte-derived, CD9 positive and Cer-enriched EVs were increased. Imipramine reduced the number of CD9 positive EVs and the number of astrocyte-derived EVs to WT levels (Fig. 6B). Furthermore, the correlation of Cer with GFAP positive EVs was reduced by 40% by Imipramine (Fig. 6C).

**Fig. 7:**
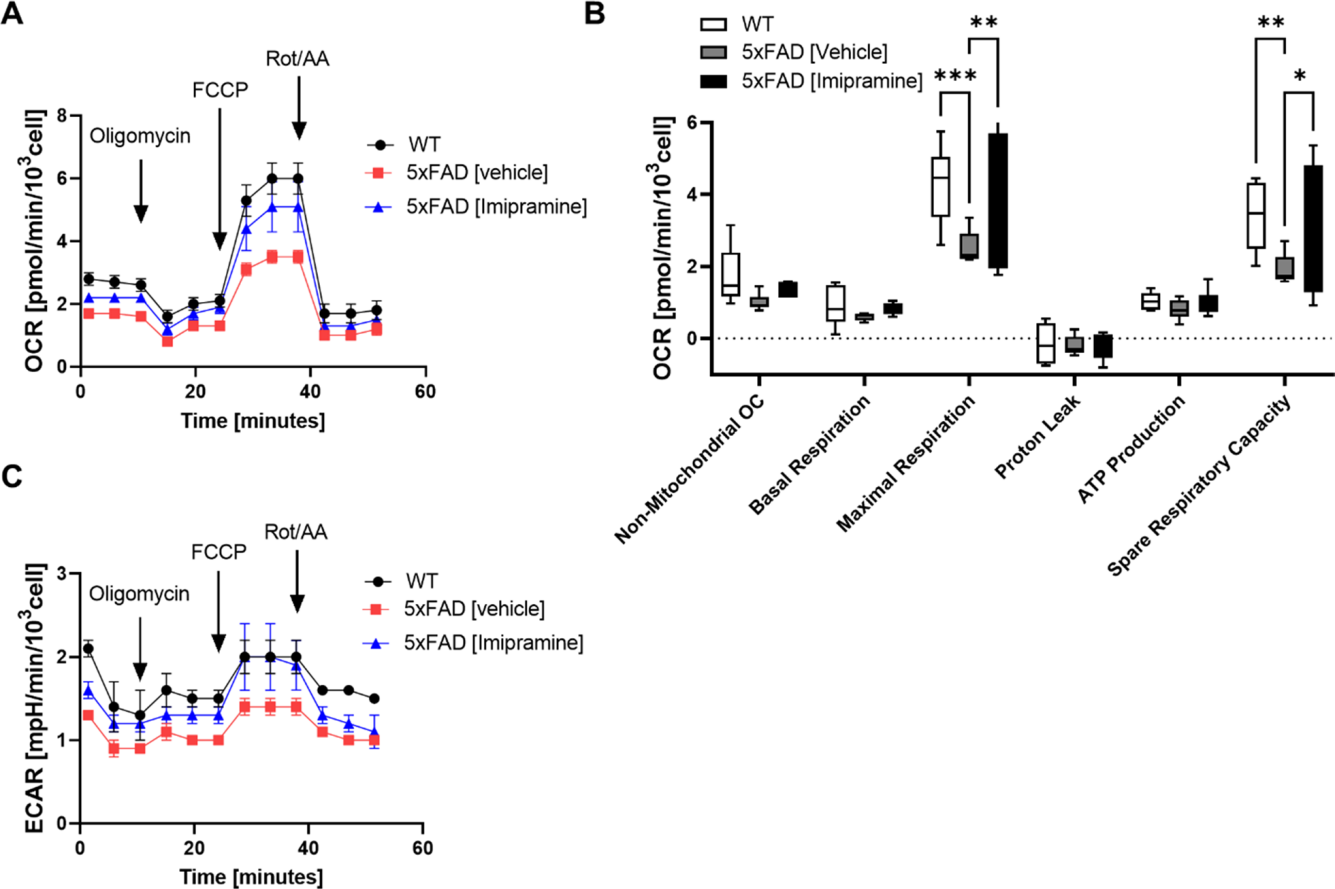
Imipramine reduces mitotoxicity of 5xFAD brain-derived EVs. **A** Bioenergetics of neuronal cells incubated with brain derived EVs of WT, 5xFAD treated with vehicle or Imipramine assessed by Seahorse Flux Analyzer using Cell Mito Stress Kit. Lines and Box and Whiskers plot represent average ± SD with each N = 4/group. **B** One-way ANOVA, Dunnett’s posthoc testing (*p < 0.05, **p < 0.01, ***p < 0.001). **C** Extracellular acidification rate (ECAR) of neuronal cells incubated with brain-derived EVs of WT, 5xFAD mice treated with vehicle or Imipramine. Lines represent average ± SD with each N = 4/group

Consistent with a proportion of EVs enriched with Cer, mass spectrometry analysis of Cer and sphingosine species of total EVs showed that Cer d18:1/18:0 and Cer d18:1/20:0 levels were elevated in 5xFAD compared to WT, which was reduced by Imipramine (Fig. 6D). We also measured Aβ_1-42_ levels in EVs and observed that Imipramine reduced Aβ levels in EVs (Fig. 6E). Furthermore, when assessing the neurotoxicity of the EVs we found that Imipramine reduced their toxicity (Fig. 6F). Lastly, we measured the impact of Imipramine on the EV-induced disruption of neuronal mitochondrial function using Seahorse analysis. Consistent with our in vitro data, EVs from 5xFAD brain compromised maximal and spare respiration capacity of neuronal cells as compared to WT, which was rescued by Imipramine (Fig. 7A–C). This data indicates that Imipramine prevents astrocytic release of mitotoxic EVs by reducing the number of Cer-enriched, CD9/GFAP positive EVs and Aβ_1-42_ levels in EVs.

### Imipramine prevents plaque deposition and neurodegeneration

**Fig. 8:**
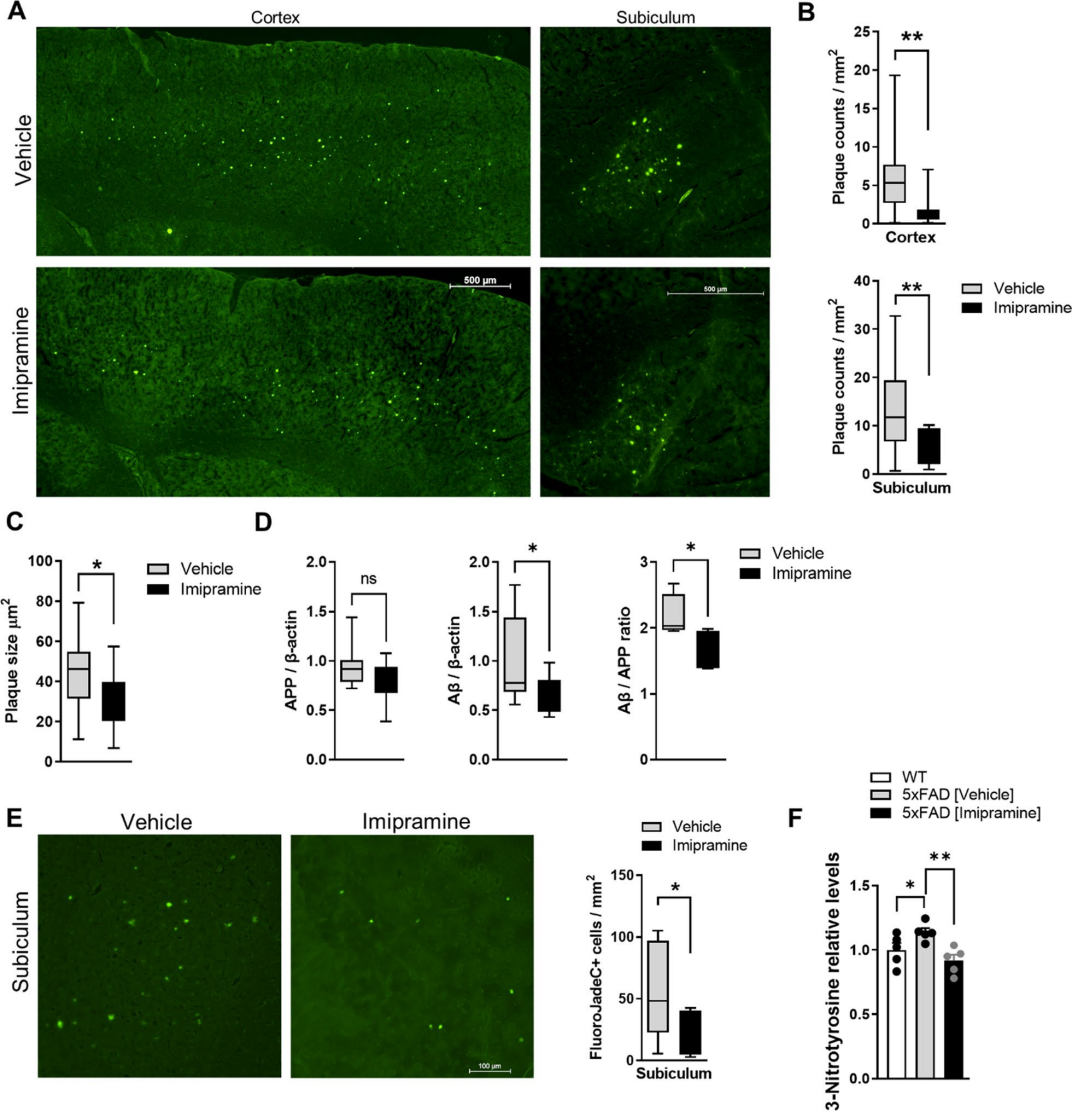
Imipramine prevents plaques deposition and neuronal cell death. **A** Representative photomicrographs of sagittal brain sections imaging the cortex and subiculum of 5xFAD labeled Aβ plaques in green. All photomicrographs were exposed and processed identically. Scale bar represents 500 µM. **B** Immunofluorescence quantification of plaques measured by plaques counts/mm^2^ and in **C** Plaques size area. **D** Western blot analysis of hippocampal homogenate stained with 6E10 antibody showing APP and Aβ levels normalized to β-actin and the ratio of amyloid Aβ/APP. Western blot membranes are shown in Additional File 1. **E** Representative photomicrographs and quantification of FluoroJadeC positive cells (Green staining) in the subiculum. Box and Whiskers plot of 4–5 animals per experimental condition, Unpaired *t*-test was applied (*p < 0.05, **p < 0.01). **F** Quantification of 3-Nitrotyrosine relative levels in pre-frontal cortex of WT, 5xFAD vehicle and Impramine groups. Bar graphs represent the mean ± SD of N = 5/group. One-way ANOVA, LSD (*p < 0.05, **p < 0.01)

Our data show that Imipramine decreased proinflammatory activation of microglia and astrocytes, as well as the secretion of Cer-enriched, mitotoxic EVs in 5xFAD mice. Previous studies reported that Imipramine improves memory symptoms in AD patients [43]. Therefore, we investigated the effect of Imipramine on Aβ plaque deposition and neurodegeneration. Statistical analysis showed a significant reduction in the plaque load between the 5xFAD treated with vehicle or Imipramine-treated mice, both in the cortex and subiculum (Fig. 8A and B). Additionally, the plaque size was reduced in Imipramine-treated 5xFAD brains (Fig. 8C). Furthermore, Aβ quantification of brain homogenates showed that Aβ levels were reduced in samples treated with Imipramine and the ratio Aβ/FL-APP was decreased implying a reduction of Aβ biogenesis or increased clearance of Aβ (Fig. 8D). Detection of degenerating mature neurons in the subiculum by FluorJadeC confirmed a protective effect of Imipramine from AD pathology (Fig. 8E). Furthermore, 3-Nitrotyrosine levels, which are indicative of oxidative stress, were reduced in the 5xFAD drug-treated group (Fig. 8F). Importantly, no differences in the drug response were found between sexes. This data suggest that Imipramine prevents Aβ-induced neurodegeneration by reducing Aβ plaques and oxidative stress indicators like 3-Nitrotyrosine levels.

## Discussion

In the brain of AD patients, the enzyme A-SMase is upregulated, and the enzymatic activity abnormally high [9]. In this work, we report that A-SMase is upregulated in microglia in response to Aβ oligomers via activation of the TLR4 receptor. Inhibition of A-SMase activity using Imipramine or ARC39 reduced microglia release of proinflammatory factors such as C1q, IL1-α and TNF-α which are critical for astrocyte phenoconversion to a reactive state. Additionally, Imipramine prevented increase of Cer levels in astrocytes and secretion of mitotoxic EVs.

Neuroinflammation is thought to play a crucial role in the development and exacerbation of AD pathology [44]. Genome-wide association studies implicate microglia-related pathways in AD [45]. One way in which microglia contribute to disease is by initiating an immune response when exposed to Aβ oligomers, which over time becomes chronic. This causes the release of proinflammatory factors that induce astrocyte activation. Previous studies on immune cells suggested that Cer generated by A-SMase is critical for cytokine release [31, 32]. It was also reported that activation of the TLR4 receptor requires Cer generation by A-SMase in immune cells [46]. Additionally, immunohistochemistry analysis in the brain of AD patients suggested that A-SMase was mostly associated with microglia [34]. However, others have shown that A-SMase activity is elevated in neuronal cells and fibroblasts isolated from AD transgenic models [11]. We found that A-SMase activity in microglia was increased by Aβ oligomers and required TLR4 receptor activation. We excluded that activation was due to TNF-α autocrine action by including an TNF-α inhibitor in the assay. Our experiment did not exclude the possibility of direct action of Aβ oligomers on A-SMase activity. As previously reported A-SMase was required for the secretion of proinflammatory factors like C1q, IL1-α and TNF-α [31, 32]. Also, in vivo inhibition of A-SMase with Imipramine reduced the number of activated microglia suggesting that the A-SMase/Cer pathway in microglia is a target for reducing neuroinflammation.

We previously discovered that reactive astrocytes are characterized by the elevation of Cer levels in the brain of AD patients and mice models of AD [16, 25, 34]. Once in the reactive state, astrocytes release a population of EVs that target neurons compromising mitochondria function [35]. These EVs are characterized by enrichment in Cer and Aβ. The high concentration of Cer in these EVs increases dramatically the mitotoxic effect of Aβ [35]. In addition, we reported that the enzyme neutral sphingomyelinase 2 (N-SMase2) played a critical role in generating these toxic EVs [47]. When inactivating N-SMase2 genetically or pharmacologically, brain EVs were lower in number and characterized by reduced levels of Cer and Aβ [47]. These findings encouraged the synthesis of additional N-SMase2 inhibitors as potential therapeutics for AD [48]. However, targeting N-SMase2 seemed to be ineffective in female 5xFAD mice, suggesting that other enzymes of the sphingolipid pathway are involved in forming these toxic EVs. While evidence of sexual dimorphism is gaining more attention in EVs research, it is poorly studied in AD. We recently found that A-SMase is associated with brain derived EVs and it is highly expressed in 5xFAD, especially female 5xFAD [27]. Concomitantly to the increase of A-SMase expression, brain-derived-EVs of female 5xFAD are characterized by the elevation of long chain Cer [27], clearly showing that A-SMase plays a critical role in Cer enrichment of EVs.

In the current study, we found that Imipramine reduced the number of EVs derived from astrocytes and diminished the content of Aβ in EVs while only modestly lowering the Cer concentration in the total population of EVs. However, Imipramine depleted Cer associated to EVs derived from astrocytes by 40%. Inhibition of A-SMase was sufficient to drastically reduce the mitotoxicity of 5xFAD brain derived EVs. Notably, EVs derived from the brain of 5xFAD show upregulation of Cer 18:1/18:0 and Cer 18:1/20:0 and mitotoxicity already at 3 months of age, before any memory deficits are detectable by behavioral paradigms.

It is important to highlight that the beneficial effect of Imipramine may arise from targeting A-SMase in several cell types and cell signaling pathways. In microglia, our data show that A-SMase is critical for the Aβ-induced secretion of proinflammatory factors through the TLR4-pathway. In astrocytes, A-SMase controls the release of large size vesicles after stimulation of ATP receptors [49]. Bianco et al., reported that activation of A-SMase was downstream to p38 MAPK cascade, which is also activated in response to TLR4 ligands [50]. In addition, they showed that the p38 MAPK cascade mobilizes A-SMase from the lysosome to the plasma membrane triggering membrane blebbing and EVs formation. While Bianco et al. did not show that cytokines induce secretion of EV or characterizes the lipid profile of these EVs, they found that A-SMase is released with these population of EVs. Other studies showed that cytokines such as Il-1β or TNF-α can induce EV secretion by astrocytes, however, these studies did not investigate the function of A-SMase or enrichment of EVs with ceramide [36, 37].

In our work, we found that CD9 positive EVs derived from astrocytes were mostly affected by proinflammatory factors and Imipramine treatment. The factors C1q, IL1-α and TNF-α triggered the secretion of a 1.8-fold increase of CD9 positive EVs from astrocytes and A-SMase inhibition reduced CD9 positive EVs and Cer levels associated with astrocytic EVs. Interestingly, CD9 positive EVs were associated with EV generated by blebbing of the plasma membrane [51]. Therefore, Cer generated by A-SMase in the plasma membrane or endolysosomal membranes in reactive astrocytes could be critical for the formation of toxic EVs. This mechanism is supported by our recent study showing that fluoxetine, another FIASMA similar to Imipramine, prevents complex formation between ceramide-enriched cellular membranes in astrocytes and Aβ [52]. While this mechanism is consistent with our data, it is also clear that neither A-SMase nor the effect of A-SMase inhibition is confined to a particular cell type. Hence, alternative mechanisms to be tested could include the effect of microglia-derived EVs on astrocytes or astrocytic EVs on or microglia. Therefore, in future studies we will further investigate the A-SMase/Cer pathway in reactive astrocytes and its critical function for toxic EV generation.

Tricyclic anti-depressants (TCAs), such as Imipramine, have been used in the treatment of the depressive symptoms in AD. Depression is one of the common comorbidities of AD that appears during the progression of the disease [53]. Treatment of AD with antidepressants has been successful not only in controlling depressive symptoms but also in rescuing the cognitive decline [43]. Moreover, anti-depressants given to AD animal models demonstrated to be effective in coping with depressive as well as cognitive symptoms [54–56]. While TCAs are known to block serotonin and norepinephrine transporters, recent studies suggest that their activity as FIASMAs reducing forebrain ceramide levels critically contributes to their anti-depressant effect [57–59]. Therefore, it is reasonable to imply the reduction of ceramide as a potential mechanism by which Imipramine contributes to mitigation of AD pathology.

In line with this proposed mechanism, partial ablation of A-SMase expression in the AD mice model reduced Aβ pathology and prevented memory impairment [11]. More recently, new approaches using different ASM inhibitors or immunotherapy with antibodies against A-SMase were shown to alleviate AD pathology, however, these studies did not address the function of A-SMase in glial activation and the secretion of neurotoxic EVs [60, 61]. FIASMAs such as Imipramine reduce Cer levels, glial activation, and the secretion of neurotoxic EVs in the brain, and they are clinically approved and immediately available for AD therapy.

In this study, we found that Imipramine prevented plaque formation in the cortex and subiculum. Furthermore, neuronal death was strongly diminished in the Imipramine-treated group. Nevertheless, the choice of FIASMA that are also tricyclic anti-depressants will need to be carefully tested. For example, long term administration of anti-depressants like escitalopram, showed to be inefficient in controlling plaques disease and even contraindicated [62]. Therefore, the synthesis or discovery of specific A-SMase blockers,will help to answer whether in vivo inhibition of A-SMase during AD pathology is an effective therapy [63].

## Conclusion

In conclusion, we report that A-SMase is activated in microglia by Aβ oligomers via TLR4 receptor. The Cer generated by A-SMase is critical for the release of C1q, TNF-α and IL1-α, which induce the reactive astrocytes phenotype. Administration of Imipramine to 5xFAD for 4 weeks prevented microglia and astrocyte activation. Furthermore, brain derived-EVs from 5xFAD mice treated with Imipramine were less mitotoxic and displayed reduction in astrocytic marker GFAP and Aβ concentration when compared to EVs derived from 5xFAD brain treated with vehicle. Our study highlights A-SMase as a target in AD to prevent microglia and astrocytes activation, and warrants synthesis and testing of brain permeable compounds, which specifically inhibit A-SMase as anti-AD therapeutic strategy.

### Supplementary Information

**Additional file 1.** **Supplementary Fig. 1**: **A** Standard curve built with known pmol of A-SMase and measured relative fluorescent units (RFU). **B** Representative experiment showing A-Smase acrivity expressed in RFU of 20 μg of microglia lysate stimulated with Aβ oligos in presence of Imipramine and TNF-α inhibitor. **Supplementary Fig. 2**: Primary cultures of microglia were incubated with 1 μM scrambled Aβ42 or Aβ42 overnight. Immunocytochemistry was performed with antibodies against Aβ42 (4G8), A-SMase (Proteintech rabbit IgG), and ceramide (mouse IgM, Glycobiotech MAB0014). **Supplementary Fig. 3**: Cryosections (cortex) were immunolabeled for Aβ42 (rabbit IgG, ThermoFisher), iNOS (mouse IgG, BDBiosciences), and Iba-1 (goat IgG, Novus). **Immunoblot images**. **A** Immunoblots showing detection of GFAP and **B** C3b protein expression. **C** β-actin was used as internal control. **D** Immunoblots showing detection of FL-APP, and Aβ bands with 6E10 antibody. **E** β-actin was used as internal control.

## Data Availability

The datasets used and/or analyzed during the current study are available from the corresponding author on reasonable request.

## Abbreviation

AD: Alzheimer’s disease
A-SMase: Acid sphingomyelinase
EVs: Extracellular vesicles
Cer: Ceramide
Aβ: Amyloid-β
TCA: Tricyclic anti-depressant
FIASMA: Functional inhibitors of A-SMase
WT: C57BL/6 wild type
IP: Intraperitoneally
PFA: Paraformaldehyde
C1q: Complement component 1
IL-1α: Interleukin 1 α
TNFα: Tumor necrosis factor α
GFAP: Glial fibrillary acidic protein
CD9: Cluster of differentiation 9
