# Supplementary material for: Inhibition of acid sphingomyelinase reduces reactive astrocyte secretion of mitotoxic extracellular vesicles and improves Alzheimer’s disease pathology in the 5xFAD mouse
Source: Acta Neuropathol Commun. 2023 Aug 21;11:135. doi: 10.1186/s40478-023-01633-7 (PMC10440899; doi:10.1186/s40478-023-01633-7)
Supplement: Supplementary file 1 — Additional file 1. Supplementary Fig. 1: A Standard curve built with known pmol of A-SMase and measured relative fluorescent units (RFU). B Representative experiment showing A-Smase acrivity expressed in RFU of 20 μg of microglia lysate stimulated with Aβ oligos in presence of Imipramine and TNF-α inhibitor. Supplementary Fig. 2: Primary cultures of microglia were incubated with 1 μM scrambled Aβ42 or Aβ42 overnight. Immunocytochemistry was performed with antibodies against Aβ42 (4G8), A-SMase (Proteintech rabbit IgG), and ceramide (mouse IgM, Glycobiotech MAB0014). Supplementary Fig. 3: Cryosections (cortex) were immunolabeled for Aβ42 (rabbit IgG, ThermoFisher), iNOS (mouse IgG, BDBiosciences), and Iba-1 (goat IgG, Novus). Immunoblot images. A Immunoblots showing detection of GFAP and B C3b protein expression. C β-actin was used as internal control. D Immunoblots showing detection of FL-APP, and Aβ bands with 6E10 antibody. E β-actin was used as internal control. [file 40478_2023_1633_MOESM1_ESM.pdf]

Supplementary Material

Supplementary Figure 1

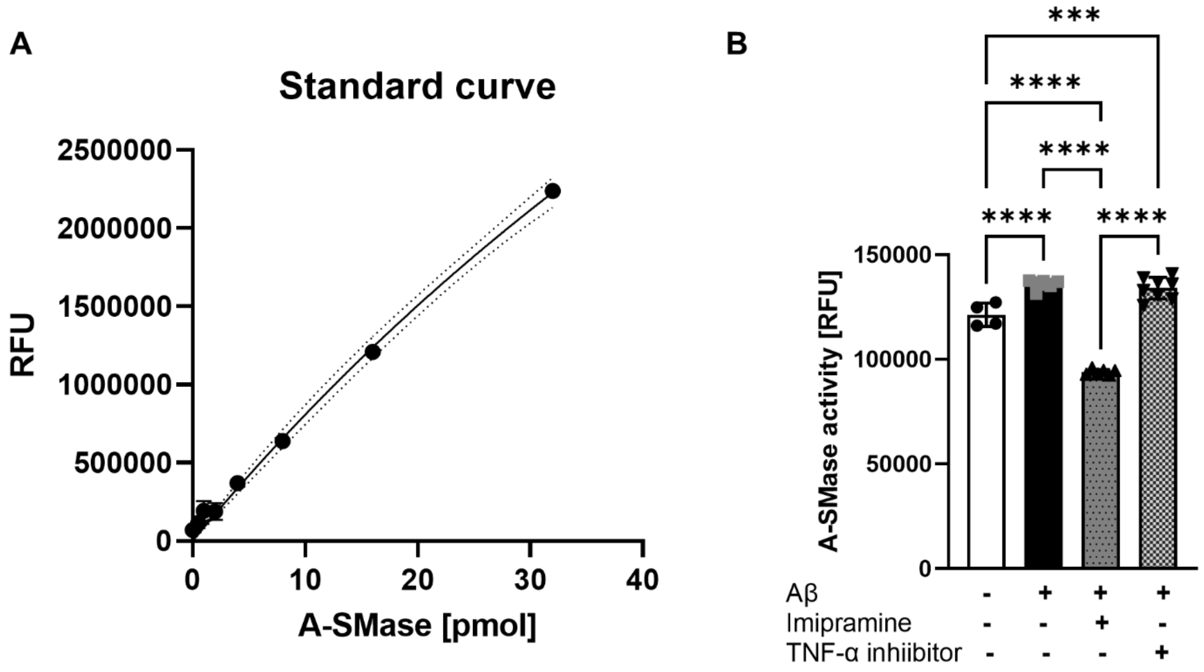

Supplementary Fig. 1: A Standard curve built with known pmol of A-SMase and measured relative fluorescent units (RFU). **B** Representative experiment showing A-Smase acrivity expressed in RFU of 20 µg of microglia lysate stimulated with Aβ oligos in presence of Imipramine and TNF-α inhibitor.

Supplementary Figure 2

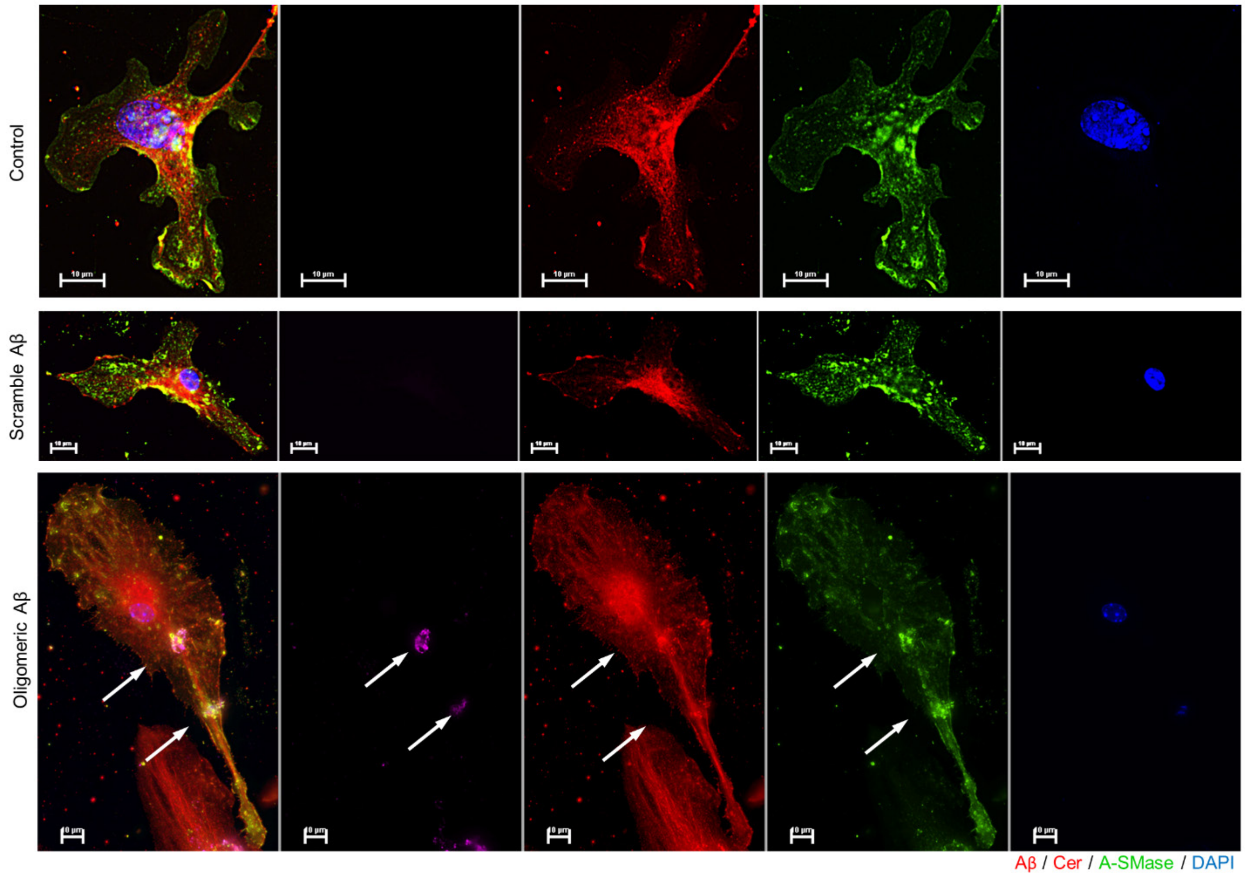

Supplementary Fig. 2: Primary cultures of microglia were incubated with 1 μM scrambled Aβ_{42} or Aβ_{42} overnight. Immunocytochemistry was performed with antibodies against Aβ_{42} (4G8), A-SMase (Proteintech rabbit IgG), and ceramide (mouse IgM, Glycobiotech MAB0014).

Supplementary Figure 3

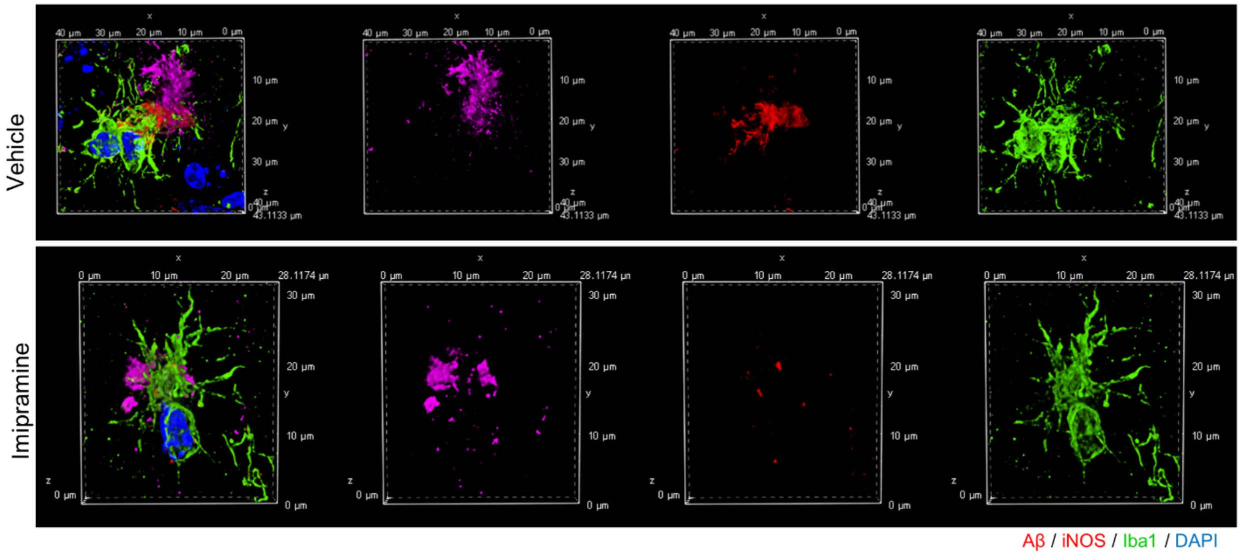

Supplementary Fig. 3: Cryosections (cortex) were immunolabeled for Aβ42 (rabbit IgG, ThermoFisher), iNOS (mouse IgG, BDBiosciences), and Iba-1 (goat IgG, Novus).

Immunoblot images

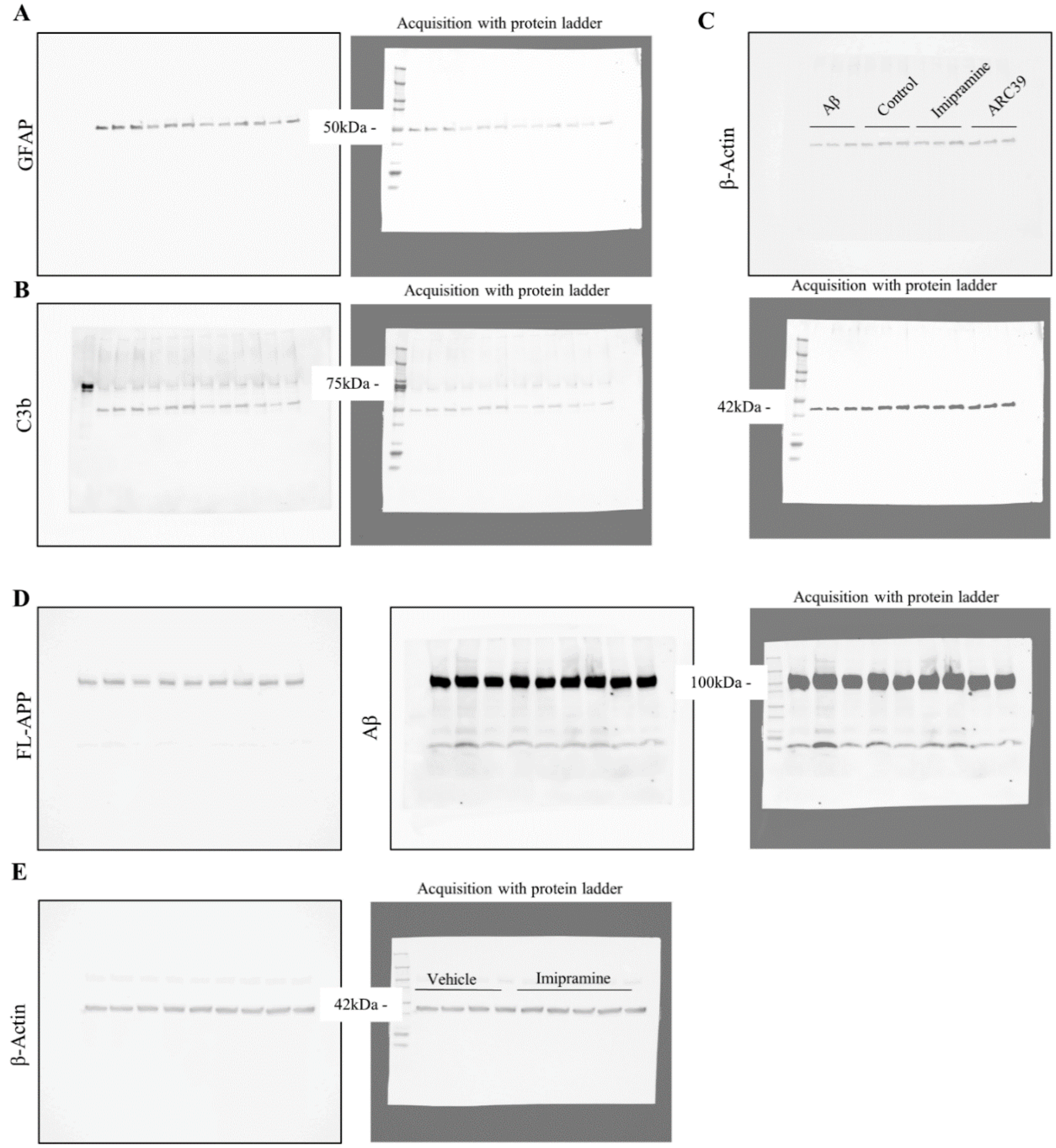

Immunoblot images. A Immunoblots showing detection of GFAP and **B** C3b protein expression. **C** β-actin was used as internal control. **D** Immunoblots showing detection of FL-APP, and Aβ bands with 6E10 antibody. **E** β-actin was used as internal control.
